# Book Review: Exploring the Interface Between Individual Difference Variables and the Knowledge of Second Language Grammar

**DOI:** 10.3389/fpsyg.2021.812334

**Published:** 2021-12-09

**Authors:** Longyang Wang

**Affiliations:** Chinese Language Department, Jiangxi Normal University, Nanchang, China

**Keywords:** second language, language grammar, individual difference variables, language, education

The demarcation between declarative (explicit) and procedural (implicit) knowledge of second or foreign language (L2) grammar seems essential to second language acquisition research and grammar instruction (DeKeyser, 2003, 2005; Pawlak, 2019) inasmuch as these two kinds of interventions are crucially impacted by various instructional techniques and how their development is manifested in different learning situations. In spite of the fact that extensive research has been conducted on the efficacy of these dichotomous teaching approaches, little research has been done on the role of individual difference (ID) variables; consequently, Mirosław Pawlak's empirically-driven monograph, titled “*Exploring the Interface Between Individual Difference Variables and the Knowledge of Second Language Grammar*,” is a substantiation to such a call in EFL classrooms to probe into the mediating effects of such ID factors as beliefs about grammar instruction (BGI), working memory (WM), grammar learning strategies (GLS), motivation, and willingness to communicate (WTC).

As to the structure of this much-needed monograph, it is organized around five chapters. The first chapter zooms in on the significance of different dimensions of L2 grammar knowledge such as form, meaning, use and relates the complexity of L2 grammar learning to transformational criteria, salience, communicative value, and the influence of the L1. It also focuses on the demarcation between explicit and implicit knowledge, concluding that “the strong interface position appears to be the most compatible with the existing teaching realities, at least in most foreign language contexts” (p. 8). The chapter then appraises and synthesizes different approaches to measure target language grammar attainment, accentuating the dire need to take into consideration the key role of ID factors. Pawlak, an influential and prolific trendsetter in GLS and grammar L2 instruction, thoughtfully encompasses BGI, WM, GLS, motivation, and WTC succinctly elaborates on the theoretical and empirical studies

Chapter Two foregrounds the pivotal role of the mediating role of ID variables in the process of teaching and learning L2 grammar with respect to implicit and explicit teaching techniques. Moreover, the chapter provides the conceptual and theoretical background for the impact of mediating the aforementioned ID factors on the knowledge of L2 grammar. Chapter 3 presents the methodology of six separate studies that scrutinized the mediating effects of the abovementioned cognitive and affective ID factors concerning explicit and implicit knowledge of the English passive. The author then clearly explains the objectives of each of the studies, the samples of participants, the pertinent data-collection instruments, and data analysis methods. Although three of the studies reported here have already been published, the rest are original and can offer plenty of food for thought. Had the author offered a composite and comprehensive model of how these cognitive and affective ID variables mutually interacted with explicit and implicit knowledge of the English passive, it would have provided the readers with the dynamic and complex nature of these variables. I suppose the author could have run Structural Equation Modeling (SEM), instead of correlations, to see the interplay of these factors on L2 grammar.

Chapter Four elaborates on the findings of the six studies separately and discusses their findings convincingly. The chapter reported that “correlation analyses and regression analyses in the six studies showed that relationships between the ID factors in question and different facets of L2 knowledge were at best moderate but mostly weak and they did not hold at all for many subscales” (p. 92). However, when the final scores of their self-assessment were added to the regression models, their predictive power indicated an improvement. Providing a wealth of information about these six innovative and opportune studies in one single chapter has convinced me to replicate these studies in China, triangulating the studies with both qualitative and quantitative data.

Chapter Five closes the monograph, provides a thorough discussion of the findings, brings to the fore the limitations inherent in these studies, offers insightful pedagogical implications, and opens up some avenues for future studies to deeply delve into how emotional and cognitive ID factors lead to the mastery of L2 grammar. The chapter also concludes that “it should be highlighted that the observed contribution of ID factors turned out to be the strongest in the case of variables that are amenable to manipulation to some extent (i.e., beliefs, motivation, and WTC)” (p. 94). The inclusion of all the instruments in the Appendix of the book motivates ardent readers to apply them in other homogenous or heterogeneous contexts.

Had the book included other grammar features and other conceived grammar attainments, not just passive L2 knowledge, the results would have been much more insightful to augment the effectiveness of L2 grammar instruction in a wide array of situations. I also expected to see a comprehensive model of how these factors interact with each other to construct productive and receptive dimensions of explicit and implicit knowledge of TL grammar. All in all, undoubtedly, this theoretically-rich, empirically-robust, and pedagogically-insightful volume provides a springboard for EFL/ESL learners, teachers, researchers, and syllabus designers who are interested in how affective and cognitive factors contribute to the attainment of L2 grammar features.

## Author Contributions

The author confirms being the sole contributor of this work and has approved it for publication.

## Conflict of Interest

The author declares that the research was conducted in the absence of any commercial or financial relationships that could be construed as a potential conflict of interest.

## Publisher's Note

All claims expressed in this article are solely those of the authors and do not necessarily represent those of their affiliated organizations, or those of the publisher, the editors and the reviewers. Any product that may be evaluated in this article, or claim that may be made by its manufacturer, is not guaranteed or endorsed by the publisher.
